# Anatomy, histology, development and functions of *Ossa cordis*: A review

**DOI:** 10.1111/ahe.12861

**Published:** 2022-09-08

**Authors:** Adam Best, Monika Egerbacher, Sophia Swaine, William Pérez, Aziza Alibhai, Paul Rutland, Valentina Kubale, Samir A. A. El‐Gendy, Mohamed A. M. Alsafy, Kerstin Baiker, Craig J. Sturrock, Catrin Sian Rutland

**Affiliations:** ^1^ School of Veterinary Medicine and Science Faculty of Medicine and Health Science, University of Nottingham Leicestershire UK; ^2^ Admin. Unit of Veterinary Medicine UMIT TIROL – Private University for Health Sciences, Medical Informatics and Technology Hall in Tirol Tyrol Austria; ^3^ Unidad de Anatomía, Facultad de Veterinaria Universidad de la República Montevideo Uruguay; ^4^ University College London Great Ormond Street Institute of Child Health London UK; ^5^ Veterinary Faculty, Institute of Preclinical Sciences University of Ljubljana Ljubjana Slovenia; ^6^ Department of Anatomy and Embryology Faculty of Veterinary Medicine, Alexandria University Egypt; ^7^ The Hounsfield Facility, School of Biosciences University of Nottingham Leicestershire UK

**Keywords:** bone, cardiac, *Cartilago cordis*, ectopic bone, *Ossa cordis*, ossification

## Abstract

This systematic review highlights the similarities and variations in *Ossa cordis* prevalence, histology and anatomical location between differing veterinary species and in humans. In addition, it also identifies associated factors such as aging and cardiovascular disease for each species in relation to functional roles and developmental mechanisms that these bone structures may play. The potential functions of *Ossa cordis* are presented, ranging from aiding cardiac contraction and conduction, providing cardiac structure, and protecting components of the heart, through to counteracting high mechanical stress. Furthermore, this review discusses the evidence and rationale behind the theories regarding the formation and development of *Ossa cordis* in different veterinary species and in people.

## 1 INTRODUCTION TO *OSSA CORDIS*

**FIGURE 1:**
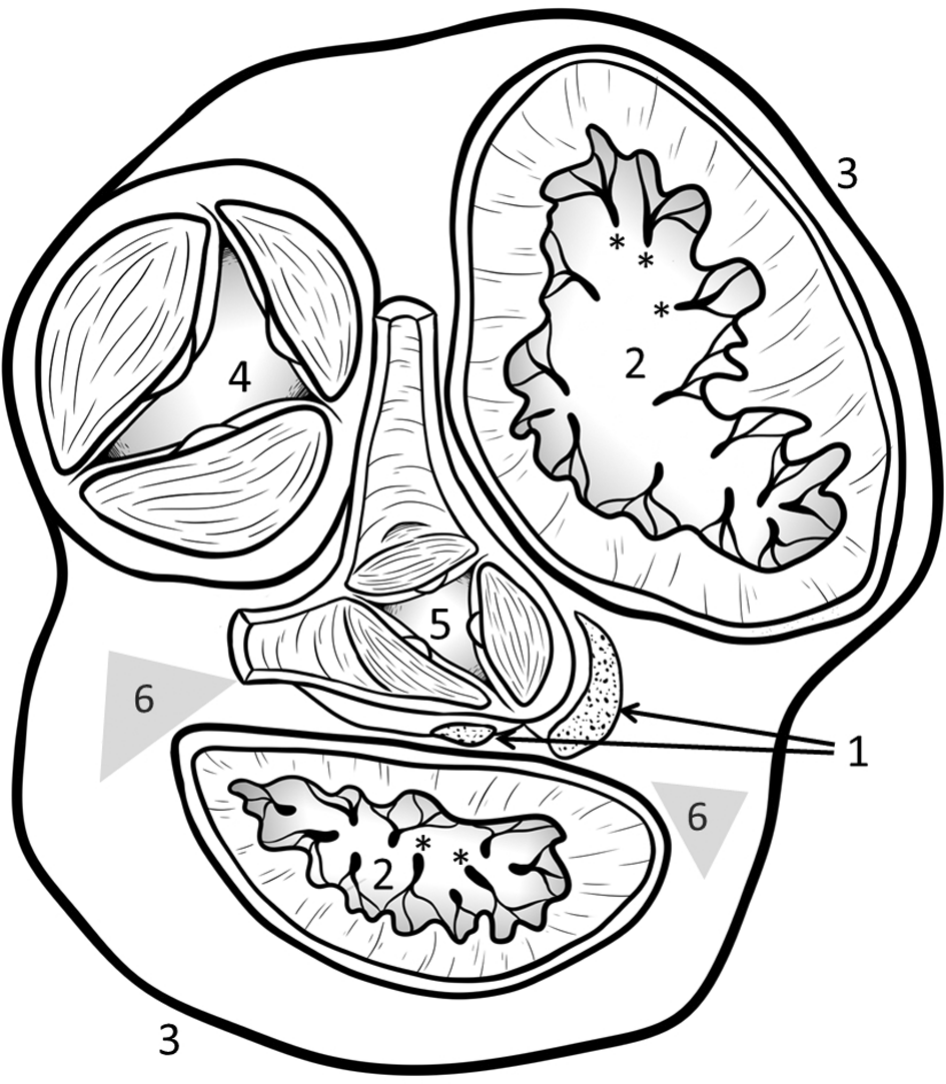
Relative position of *Ossa cordis* in the heart, dorsal aspect with of *ventriculi cordis* with the atria removed. 1. Location of *Ossa cordis* or *Cartilago cordis* (when present). 2. *Ostium atrioventriculare* (dextrum et sinistrum) surrounded by * *chordae tendinae*. 3. *Ventriculus cordis*. 4. *Ostium trunci pulmonalis* and 5. *Ostrium aortae*, both of which are surrounded by *anulus fibrosus* attaching to valve systems. 6. Relative locations of the *Trigona fibrosa*, dense connective tissue, often observed directly adjacent to *Ossa cordis/Cartilago cordis*.Adapted from (Habel et al., 2018)

In the 1800s, it was thought the *Ossa cordis* (cardiac bone) was a product of calcification in the aortic fibrous ring area (Bichat, 1819), but by 1888 it was recognized as bone tissue formed in a cartilaginous model (Vaerst, 1888). *Ossa cordis* are located within the cardiac skeleton, a fibrous structure which acts to maintain the heart's shape during systole and help ensure cardiac contraction efficiency (Habermehl & Schmack, 1986; Schmack, 1974). The cardiac skeleton is made up of two trigones, left and right‐sided, each incorporating an atrioventricular ring (Figure 1). The trigones contain fibrocartilage, hyaline cartilage and, on occasion, a bone (or less frequently bones) known as *Ossa cordis* may be present (Dyce, Sack, & Wensing, 2017; Nasoori, 2020). They are often referred to as heterotopic (abnormal growth of bone in the non‐skeletal tissues) or ectopic bones (ossification of tissues outside their usual origins). When more than one os cordis is present they are differentiated as the right‐sided, often larger os cordis dextrum, and the left‐sided, smaller os cordis sinistrum. *Ossa cordis* have been described in a limited number of species, as described in detail in this review.

In human cardiac anatomy, in addition to trigones and atrioventricular rings, the heart has a cardiac fulcrum (Figure 1). Works by Trainini and coauthors elucidated the value of this structure in humans and proposed its function and importance once they had observed the attachment of the continuous myocardium to the fulcrum and naming the structure the ‘cardiac fulcrum’ (Trainini et al., 2021; Trainini et al., 2022). They proposed that the fulcrum, a thickening at the base of the aorta made up of a collagen matrix, is essential for anchoring the myocardial band allowing the band to contract and relax, maintaining efficient cardiovascular blood flow (Trainini et al., 2021; Trainini et al., 2022). In the ageing human heart, calcium deposits have been reported in the right and left fibrous trigones (Tohno et al., 2007; Trainini et al., 2021; Trainini et al., 2022). Whilst the presence of *Ossa cordis* occurs mainly in larger species, cartilage within the cardiac skeleton (*Cartilago cordis*), which does not appear to develop into bone, has been observed in many species including hamsters, snakes, chickens, quails, chinchillas, iguanas, terrapins, horses, dogs and rhinoceros (Duran et al., 2004; Erdogan, Lima, & Perez, 2014; Jurado et al., 2006; Lopez et al., 2003; Lopez, Duran, & Sans‐Coma, 2000; Lopez, Fernandez, Duran, & Sans‐Coma, 2001; Schmack, 1974; Warchulska et al., 2016; Young, 1994). This review summarizes the literature available regarding the *Ossa cordis* in different mammalian species.

## 2 MATERIALS AND METHODS

A systematic review of the literature was undertaken across three databases (Nusearch, CAB abstracts and PubMed) in addition to google scholar and general internet searches. A total of 31 relevant papers were collected and evaluated within this review (Figure S1.). Anatomical nomenclature followed the *Nomina Anatomica Veterinaria* (ICVGAN., 2017).

Literature review inclusion criteria: Original research documenting the presentation, formation and function of *Ossa cordis*; all species included: written in English, German or French. Exclusion criteria: Review papers; written in languages other than English, French or German. No date exclusions were made.

### Keyword searches

Os* cordis; Os cordis AND heart anatomy; Os* cordis AND histology; Os* cordis AND cardiac anatomy; Osteocyte AND heart; Osteocyte AND cardiac skeleton; Osteocyte AND myocardium AND cardiac skeleton; Osteoblast AND heart; Osteoblast AND heart AND cardiac skeleton; Fulcrum AND osteocyte; Fulcrum AND heart; Fulcrum AND osteocyte AND heart

### 2.1 *Ossa cordis* Presentation

When present, *Ossa cordis* are usually associated with the atrioventricular rings and cardiac septa; however, this bone has shown a great deal of variation between species, and even individuals, in relation to its presence and number size, shape and position (Tables 1 and 2). Most animals presenting with *Ossa cordis* reside within the *Bovidae* family, within the order Artiodactyla (cattle, water buffalo, sheep, goats and antelope). The closely related *Cervidae*, *Giraffidae* and *Camelidae* families, within the Artiodactyla order also contain species with *Ossa cordis*.

**TABLE 1:** Lengths, widths and depths and overall prevalence of *Ossa cordis* in all species with *Ossa cordis*.

Species, bone	Length (mm)	Width (mm)	Depth (mm)	Prevalence % (total n studied)	Reference
Buffalo, dextrum	52 40–55	13 20–30, 2–6 (base, apex)	— —	93% (15) 83% (6)	(Daghash & FarghaliI, 2017) (David, 1937)+
Buffalo, sinistrum	23 23–30	4 —	— —	93% (15) 83% (6)	(Daghash & FarghaliI, 2017) (David, 1937)+
Camel, dextrum	—	—	—	100% (10)	(Balah et al., 2014)
Camel, dextrum	—	—	—	100% (40)	(Hegazi, 1954)
Cat, dextrum	—	—	—	30% (63)	(Liu et al., 1975)
Cattle, dextrum	40.85 30.92 51.00 $\bar{x}$ = 40.92	7.49, 18.36 5.25, 10.99 4.00, 8.40 $\bar{x\;}$= 5.58, 12.58 (cranial, caudal)	— — —	100% (40) 100% (40) 100% (8)	(Pour, 2004) (Pour, 2004) (James, 1965)
Cattle, sinistrum	19.95 17.35 18.00 $\bar{x\;}$= 18.43	9.75 7.28 11.60 $\bar{x}$ = 9.54	— — —	100% (40) 100% (40) 37.5% (8)	(Pour, 2004) (Pour, 2004) (James, 1965)
Chimpanzee	6.1	6.0	5.0	19% (16)	(Moittie et al., 2020)
Deer (white tailed)	20 18.7–22.4	5 —	— —	40% (10) 100% (84)	(Rumph, 1975) (Long & Smart, 1976)
Deer (species unknown)	24	6	—	100%? (413)	(Dupuy, 2011)
Dog, dextrum	—	—	—	73% (11)	(James & Drake, 1968)
Elephant, dextrum	95	—	—	33% (3)	(Endo et al., 2005)
Elephant, sinistrum	80	—	—	33% (3)	(Endo et al., 2005)
Giraffe, dextrum	—	—	—	100% (1)	(Perez et al., 2008)
Goat, dextrum	19	5.2*	2.3	44% (50)	(Mohammadpour & Arabi, 2007)
Sea lion, dextrum	16	42	7	9% (11)	(Yoshida et al., 2022)
Sheep, dextrum	10–15	1–2	1–2	100% (25)	(Frink & Merrick, 1974)
Sheep, dextrum	18.1	5.7*	2.3	52% (50)	(Mohammadpour & Arabi, 2007)
Sheep, dextrum	30	5	—	100% (1)	(Massari et al., 2021)
Sheep, sinistrum	5.5	5	—	40% (25)	(Frink & Merrick, 1974)+
Horse, dextrum	—	—	—	100% (1)	(Matsuda et al., 2010)
Otter, dextrum	1.5–5	—	—	40% (30)	(Egerbacher et al., 2000)

All measurements were expressed as a mean quoted directly from the manuscripts. +measured from a scaled image.? = although 413 hearts were shown, it is not known whether this represented every heart collected. *caudal width only, no values provided for cranial or median. $\bar{x}\;$= mean

**TABLE 2:** Prevalence of *Ossa cordis* in different age groups within differing species

Species (age)	Bone prevalence as % total n (total n studied)		Reference
Os cordis dextrum			
Camel (7–9 years)	100% (10)		(Balah et al., 2014)
Cat (Adult)	30% (63)		(Liu et al., 1975)
Chimpanzee (10–59 years)	18.8% (16)		(Moittie et al., 2020)
Deer, white tailed (6 months‐4.5 years)	40% (10)		(Rumph, 1975)
Deer, white tailed (1–2 years)	100% (48)		(Long & Smart, 1976)
Deer, white tailed (2 years+)	100% (36)		(Long & Smart, 1976)
Deer, unspecified (1–14 years)	100% (413)		(Dupuy, 2011)
Dog (≤9 weeks)	33.3% (3)		(James & Drake, 1968)
Dog (3 years)	0% (1)		(James & Drake, 1968)
Dog (≥3.5 years)	100% (7)		(James & Drake, 1968)
Giraffe (unknown age)	100% (1)		(Perez et al., 2008)
Goat (1.5–2 years)	44% (50)		(Mohammadpour & Arabi, 2007)
Horse (4 years)	100% (1)		(Matsuda et al., 2010)
Otter (Juvenile, <1 years)	33.3% (3)		(Egerbacher et al., 2000)
Otter (Sub‐adult, 1–2 years)	11.1% (9)		(Egerbacher et al., 2000)
Otter (Adult, >2 years)	84.6% (13)		(Egerbacher et al., 2000)
Otter (Unknown Age)	40.0% (5)		(Egerbacher et al., 2000)
Sea lion (pup and subadult)	0% (5)		(Yoshida et al., 2022)
Sea lion (adult)	16.6% (6)		(Yoshida et al., 2022)
	**Os cordis Dextrum**	**Os cordis Sinistrum**	
Buffalo (1 month‐3 years)	0% (1) 0% (1)	0% (1) 0% (1)	(Daghash & FarghaliI, 2017) (David, 1937)
Buffalo (3 years+)	100% (14) 100% (6)	100% (14) 100% (6)	(Daghash & FarghaliI, 2017) (David, 1937)
Cattle, Holstein (<2 years)	100% (40)	80.0% (40)	(Pour, 2004)
Cattle, Iranian (<2 years)	100% (40)	20.0% (40)	(Pour, 2004)
Cattle, beef, no breed stated	100% (8)	37.5% (8)	(James, 1965)
Elephant (21–38 years)	0% (2)	0% (2)	(Endo et al., 2005)
Elephant (56 years)	100% (1)	100% (1)	(Endo et al., 2005)
Sheep (Fetus)	0% (5)	0% (5)	(Nabipour & Shahabodini, 2007)
Sheep (4 months)	100% (1)	0% (1)	(Massari et al., 2021)
Sheep (Unknown Age)	100% (25)	40.0% (25)	(Frink & Merrick, 1974)
Sheep (1.5–2 years)	52% (50)	0% (50)	(Mohammadpour & Arabi, 2007)

### 2.2 Cattle

**FIGURE 2:**
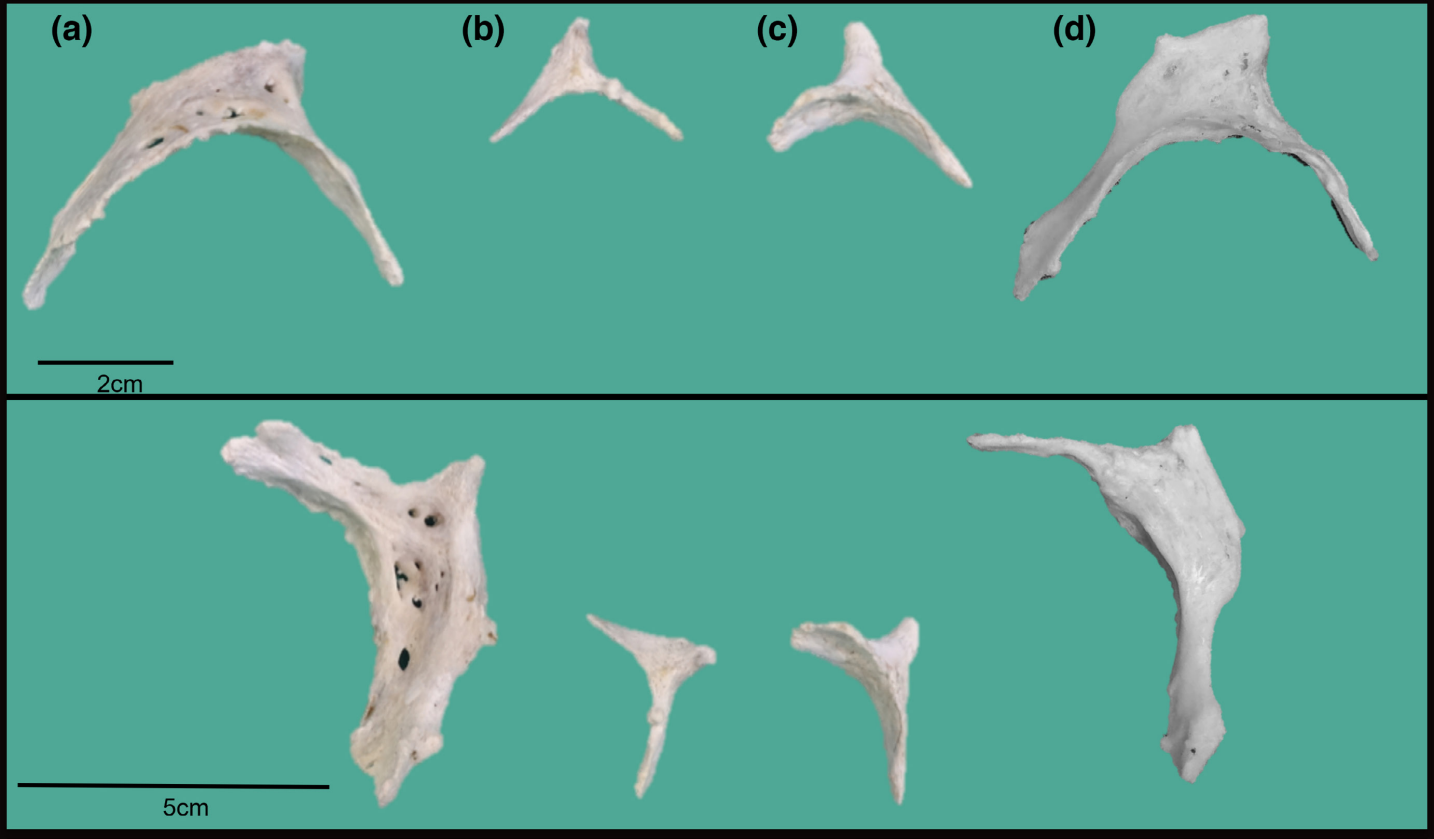
Cattle *Ossa cordis* photographs from two different aspects. Adult os cordis (a) dextrum and (b) sinistrum from one heart and (c) sinistrum and (d) dextrum from another individual

In cattle (*Bos taurus*), *Ossa cordis* (Figure 2) have been well documented, and each individual may have one or two cardiac bones (Habermehl & Schmack, 1986; James, 1965; Pour, 2004). Os cordis dextrum was found in 100% (*n* = 80) of the hearts investigated (Pour, 2004) and were consistently located on the right side of the heart near the interventricular and interatrial septa, beneath, and extending into, the right atrioventricular ring (James, 1965). With reference to the conduction system, the os cordis dextrum was present above the bundle of His and opposite the atrioventricular node (AVN) (James, 1965). The cranial aspect of the bone sat just below the base of the aorta and the caudal aspect of the bone extended with two rami towards the coronary sinus (James, 1965; Pour, 2004). Cattle may also have an os cordis sinistrum, found in 37.5% of beef hearts (*n* = 3 from 8) (James, 1965); 80% of Holstein breeds (*n* = 32 from 40) and 20% of native Iranian cattle (*n* = 8 from 40) (Pour, 2004). The os cordis sinistrum location was relatively consistent across individuals, inserting into the left atrioventricular ring. Its average length was 18.43 mm, smaller than os cordis dextrum at 42.59 mm (James, 1965; Pour, 2004). One study suggested that the two *Ossa cordis* were always connected by cartilage (James, 1965), however in a more recent study no cartilaginous connection was reported (Pour, 2004).

### 2.3 Water buffalo

In Philippine water buffalo (*Bubalus bubalis*) 93% of hearts had two *Ossa cordis* [*n* = 14 from 15 (Daghash & FarghaliI, 2017)]. The os cordis dextrum was comparatively longer than observed in cattle, traversing between, and inserting into, the right and left atrioventricular rings. The os cordis sinistrum also differed from cattle, rather than inserting into the left atrioventricular ring it was located below the left coronary artery. Ossa cordis dextrum and sinistrum were visible in six adult (aged 7–19 years) water buffalo, with no *Ossa cordis* in a 1.5‐year‐old, where a *Cartilago cordis* was present (David, 1937). Microscopic analysis indicated bone morphology in the aortic fibrous ring, similar in size to cattle. In 5–12 years old (*n* = 10) an os cordis was referred to (no measurements or numbers) at the base of the aortic fibrous ring in each individual (Maguigad & Balagan, 2021).

### 2.4 Sheep

In sheep (*Ovis aries*), os cordis dextrum measuring 10‐15 mm (*n* = 25) was present in all hearts, situated deep within the interatrial septum. Additionally, an os cordis sinistrum was found within the left atrioventricular ring in 10 of the hearts (Frink & Merrick, 1974). Cartilaginous connections were not found between the two bones (where present) in any of the hearts. Interestingly, the os cordis dextrum consistently interrupted the normal route of the bundle of His causing it to divert under the bone. The ovine os cordis sinistrum inserted into the *Cuspis septalis* of the *Valva atrioventricularis sinistra* rather than the *Cuspis* parietalis as observed in cattle (Frink & Merrick, 1974). In 50 Lori‐bakhtiari sheep, os cordis dextrum was found in 52% of hearts, with an average length of 18.1 mm, located in a similar region to previous findings in cattle, beneath the septal cusp of the tricuspid valve (Mohammadpour & Arabi, 2007). Interestingly, in this sheep breed no *Ossa cordis* sinistrum were observed. A third bone was discovered in 17.9% of right atria investigated using radiography (*n* = 10 of 56; [Gopalakrishnan, Blevins, & Van Alstine, 2007]). Unfortunately, this study only investigated the right atria from sheep; therefore, it is possible that the third bone was a fragment of the os cordis dextrum as previously described (Frink & Merrick, 1974), or that it was not associated with *Ossa cordis* but was part of the atrial myocardium. A more recent investigation using computed tomography additionally identified a single os cordis in a four‐month‐old lamb (Massari, Ferreira‐Silva, Riceti‐Magalhães, Souza‐Silva, & Miglino, 2021).

### 2.5 Goat

In a study of 50 native goat hearts [*Capra aegagrus* hircus; (Mohammadpour & Arabi, 2007)], 44% of adult hearts presented with an os cordis dextrum. The bone was found in a similar position to the ovine os cordis dextrum, near the interventricular and interatrial septa. The os cordis dextrum in goats was thinner and more elongated and 0.9 mm longer than observed in sheep. Similar to sheep, no os cordis sinistrum was found in any of the hearts (Mohammadpour & Arabi, 2007).

### 2.6 Antelope

*Ossa cordis* have also been discovered within antelopes, which also reside within the *Bovidae* family. A single os cordis, sometimes two, were also present in 307 hearts from four species of deer (see deer section below) and two antelope species. *Antilope cervicapra* (blackbuck) and *Boselaphus tragocamelus* (nilgai) (Rodgers, George, & Bell, 2004). This conference abstract did not detail the numbers, prevalence or bone sizes, but noted *Ossa cordis* were generally only observed in mature animals.

### 2.7 Deer

The *Cervidae* family, within the order Artiodactyla, also contains species with *Ossa cordis*. *Ossa cordis* in deer have been described in many species, with size varying across species, breed, age and size of the animal. In the white‐tailed deer, *Odocoileus virginianus*, a single os cordis was described in 4 out of 10 hearts, averaging 20 mm in length and 5 mm wide (Rumph, 1975), fairly comparable to sheep and goats. *Ossa cordis* from Wisconsin (*n* = 26) male white‐tailed deer were greater in size than both Wisconsin female deer and both male and female deer from Texas (*n* = 45), but adult *Ossa cordis* measurements remained similar (Long & Smart, 1976). In contrast, yearling males had larger *Ossa cordis* than females of the same age. Variation was observed in *Ossa cordis* structure. A single os cordis, sometimes two, were also present in 307 hearts from six artiodactyl species (also see the section on antelopes) including four deer: *Axis axis* (chital/spotted deer), *Cervus nippon* (sika deer), *Dama dama* (fallow deer) and *Odocoileus virginianus* (white tailed deer), which were generally present in mature individuals (Rodgers et al., 2004). A non‐peer‐reviewed book showed that 413 deer hearts, hunted in France, had *Ossa cordis* (Dupuy, 2011). Prevalence was unavailable as the total number of individuals collected was not reported, but the author stated average bone length was similar to sheep and goats at 24 mm. In *Ozotoceros bezoarticus* (pampas deer), *Ossa cordis* were not detected in 11 adults and 9 young (Vazquez, Dos Santos, Pérez, Artigas, & Sorriba, 2019).

### 2.8 Giraffe

During an anatomical investigation of a giraffe (*Giraffa camelopardalis rothschildi*) heart, a single os cordis was reported within the right trigone of the cardiac skeleton, whilst the left fibrous trigone contained a small structure composed of hyaline cartilage (Perez, Lima, Pedrana, & Cirillo, 2008). Thus, confirming the presence of an os cordis within another member of the order Artiodactyla, but in the *Giraffidae* family.

### 2.9 Camel

In the dromedary camel (*Camelus dromedaries*: order Artiodactyla, family: *Camelidae*) a single os cordis was present in all 10 hearts investigated, in a comparable position to cattle os cordis dextrum (Balah, Bareedy, Abuel‐atta, & Ghonimi, 2014). In a further 40 specimens, every heart had a single os cordis in the right half of the aortic fibrous ring, described as transversely elongated, narrow triangle, with the cranial half more cartilaginous and the caudal region broader and more ossified (Hegazi, 1954).

### 2.10 Dog

Within the order Carnivore, the dog, cat, otter and sea lion have shown evidence of *Ossa cordis*, making this order the second largest in terms of Ossa cordis presence after Artiodactyla. Following post‐mortems in sudden death dogs (*Canis lupus familiaris*, order: Carnivore*)*, cardiac bone presented in 8 of 11 (73%) Doberman pinscher hearts (James & Drake, 1968). The bones were located within the central fibrous body, adjacent to the bundle of His and AVN; however, no observations were made on the shape or size of the bone (James & Drake, 1968).

### 2.11 Cat

Following cardiovascular disease (non‐suppurative endocarditis and myocarditis) 58 of the 63 cat (*Felis catus*; order: Carnivore) hearts had islands of cartilage present within the central fibrous body, and in a further 19 hearts bone was also found in this region (Liu, Tilley, & Tashjian, 1975). Observations regarding the sizes, positions and shapes of osseous/cartilaginous foci were not noted.

### 2.12 Otter

Another species with evidenced *Ossa cordis* is the otter (*Lutra lutra*; Figure 5), which also resides within the Carnivora order. In some hearts no bone was identified, in others only cartilage was present, whilst other hearts exhibited, one, two or more *Ossa cordis* [total *n* = 30 (Egerbacher, Weber, & Hauer, 2000)]. The bones measured 1.5‐5 mm in length, had irregular sizes and shapes, and the authors noted this was to a greater degree than reported in other species. The bones were consistently found within the cardiac skeleton trigones but other than this basic trend, the location varied widely between individual hearts (Egerbacher et al., 2000). Bone pieces beyond the typical os cordis dextrum and sinistrum were identified on X‐rays, although their exact locations were not all demonstrated, it was of interest that the number varied between individuals (Egerbacher et al., 2000). It was hypothesized that this variation was unusual in comparison to other mammals, but it may be due to the unusual cone shape of the otter heart.

### 2.13 Sea lion

The most recent species discovered to have *Ossa cordis* and *Cartilago cordis* was the Steller sea lion (*Eumetopias jubatus*; order Carnivora), the only pinniped with confirmed *Ossa cordis* so far. From the two pups, 3 subadults and 6 adults investigated, one pup and three adults had *Cartilago cordis* and one adult had an *Ossa cordis* containing marrow in the right fibrous trigone (Yoshida et al., 2022).

The final three orders containing mammals with *Ossa cordis* are Perissodactyla, Proboscidea and Primates, each containing just one species where the bone has been documented.

### 2.14 Horse

Cardiac cartilage has been found consistently in horses (*Equus caballus*; Perissodactyla order, *Equidae* family) over five years of age, however, in a four‐year‐old which died following cardiac arrhythmias, multiple bone foci were observed within the left atrioventricular valve cusps (Matsuda et al., 2010). Multifocal bone and cartilaginous foci were also observed throughout the cardiac skeleton but were not specifically designated as *Ossa cordis*.

### 2.15 Elephant

Within the Proboscidea order, the Elephantidae family have been shown to exhibit Ossa cordis. One of the most historical reports of elephant Ossa cordis was by Galen nearly 2000 years ago (Salas, 2014), and although there have since been discussions over whether he actually saw it, later studies have supported his assertions. Later Retzer, and then King and co‐authors reported the absence of any Ossa cordis in a total of one female adult (40 years old) Asian elephant (Elephas maximus), one adult (43 years old) male Asian elephant, and one young unspecified species young elephant (King, Burwell, & White, 1938; Retzer, 1912). Retzer checked for the bone by inserting a needle into the heart, so the findings may not have been accurate. A later study examining three Asian elephant hearts noted that two Ossa cordis were found in one of the hearts (Endo et al., 2005). The position of the bones was not reported but, the bones were stated as 80 and 95 mm, respectively. The causes of death for these individuals (except Galen where it is not known) were unrelated to any heart condition and no evidence of previous cardiovascular disease was reported in the studies.

### 2.16 Chimpanzee

**FIGURE 3:**
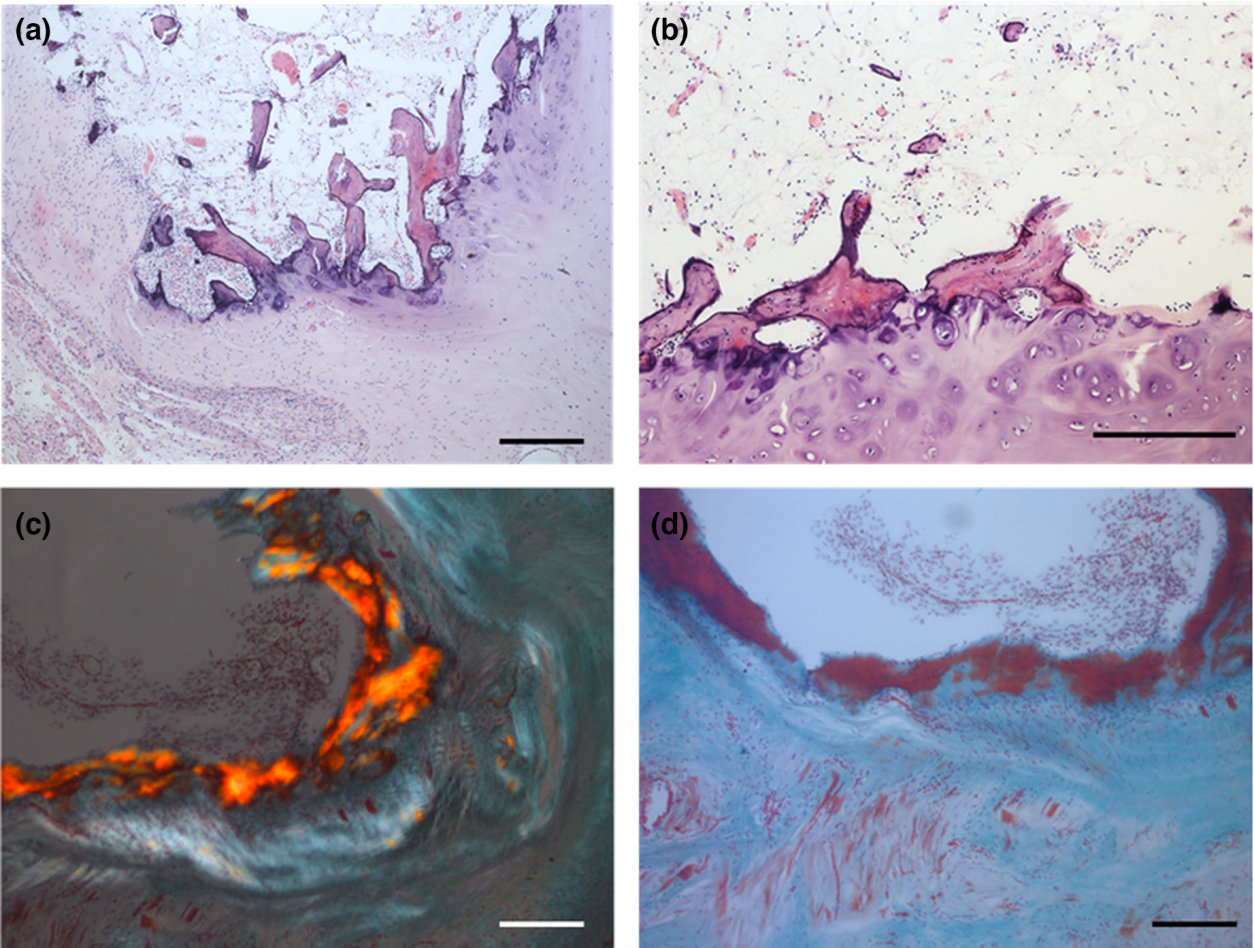
Chimpanzee *Ossa cordis* histology. Areas of lamellar bone with adipose marrow, alongside ectopic hyaline cartilage and, fibrovascular stroma, blood vessels, small numbers of haematopoietic cells and osteocytes. Photomicrographs of (a, b) haematoxylin and eosin, (c) picrosirius and (d) Masson's trichrome. Scale bars represent 200 μm. Stains conducted using the samples and methods as published previously (Moittie et al., 2020)

**FIGURE 4:**
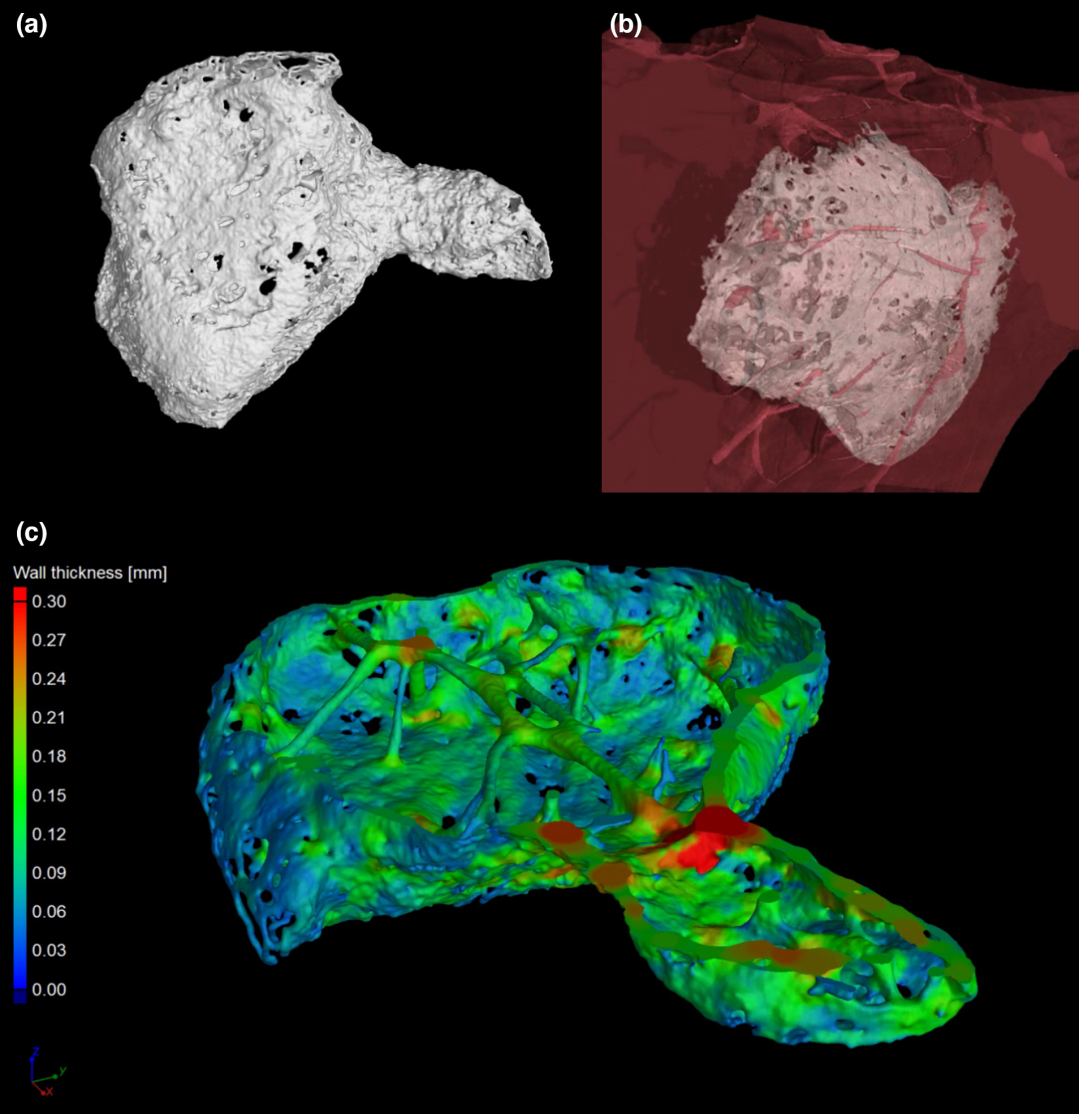
High‐resolution X‐ray computed microtomography images of chimpanzee *Ossa cordis*. 3D rendered specimens (a, b) and one with local thickness measurements (c). Data were visualized using VGStudioMAX v2.2 software (https://www.volumegraphics.com). Samples and methods used were as described previously (Keane, Paul, Sturrock, Rauch, & Rutland, 2017; Moittie et al., 2020)

Within the Primates order, the chimpanzee (*Pan troglodytes*; Figures 3, 4 and Video 1), exhibited a single os cordis present in 3 of 16 hearts (Moittie et al., 2020). The position of the bone within the trigonum fibrosum was similar to that in cattle and camels, but the bone was smaller (length $\bar{x}$ = 6.1 mm). Despite these similarities with larger mammal *Ossa cordis* it should be noted that all three chimpanzee hearts had idiopathic myocardial fibrosis, indeed the level of fibrosis was a factor associated with the presence of os cordis (Moittie et al., 2020). Therefore, rather than being anatomically neutral, as is the case with other larger mammals, the *Ossa cordis* development in chimpanzees is possibly correlated to chronic, degenerative cardiovascular disease.

**FIGURE 5:**
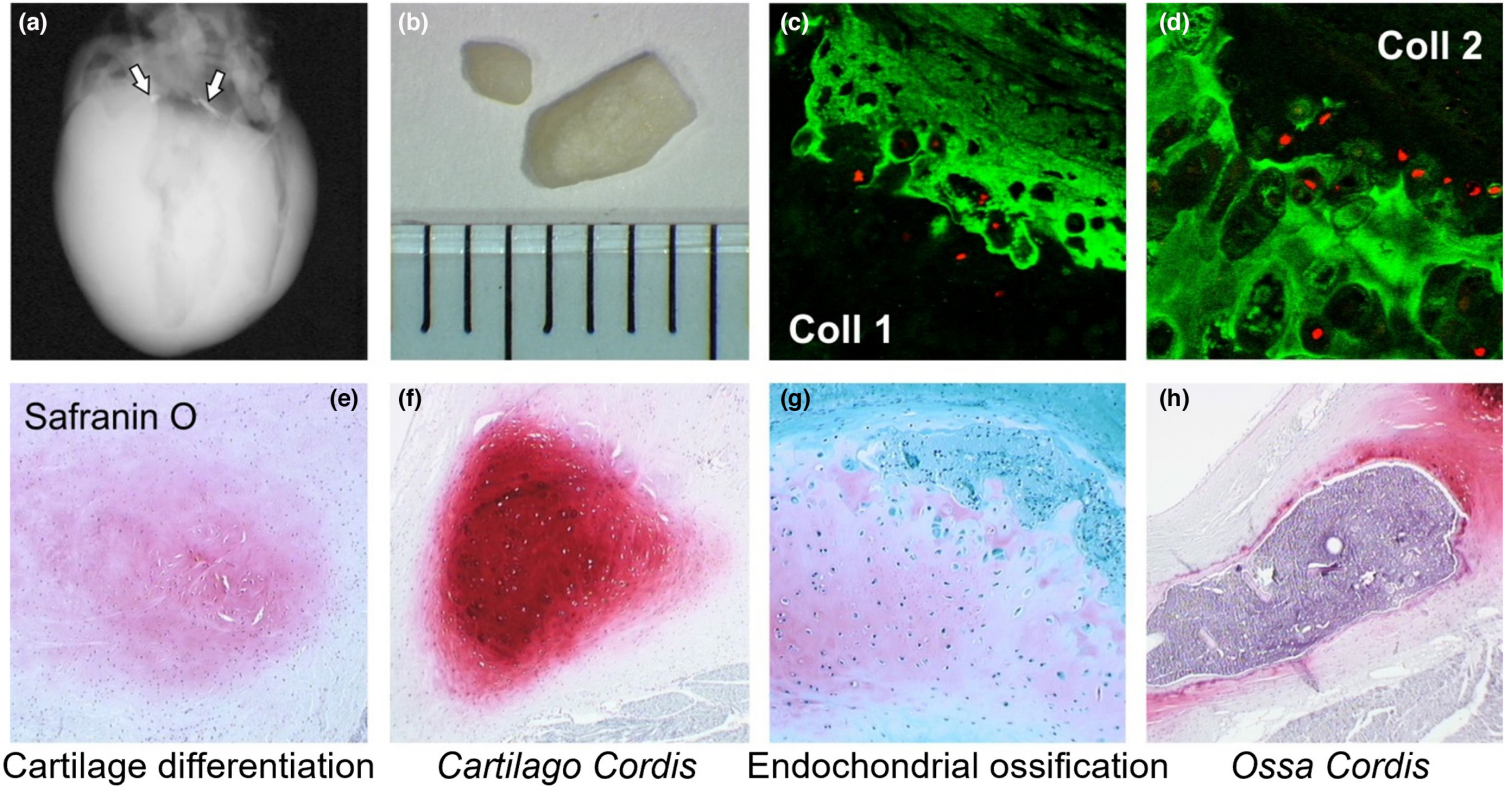
Otter *Ossa cordis* anatomy and histology. (a) *Ossa cordis* X‐ray location and (b) gross anatomy and immunohistoflourescence showing (c) collagen type I and (d) collagen type II expression. Safranin O histological staining showing (e) cartilage differentiation, (f) *Cartilago cordis* histology, (g) endochondrial (EC) ossification and (h) *Ossa cordis* histomorphology. Samples and methods as previously described (Egerbacher et al., 2000)

### 2.17 Summary of *Ossa cordis* number, location and anatomy in differing species

The usual position of os cordis dextrum across all species was on the right side of the heart, with the bone associated with the right atrioventricular ring. There was also an obvious relationship between the *Ossa cordis* and conduction system of the heart. Where an os cordis sinistrum was present, it was always smaller than os cordis dextrum and, in most species, associated with the left atrioventricular ring. Variation in the presence of os cordis sinistrum was also noted within other species, in cattle the os cordis sinistrum was only present in 20% of Iranian breeds in comparison to 80% of Holstein cattle (Pour, 2004). Differences between breeds were also noted in sheep where os cordis sinistrum was found in 40% (10 out of 25) of sheep hearts [breed unknown; (Frink & Merrick, 1974)] but was not found in any of 50 Iranian sheep breed hearts (Mohammadpour & Arabi, 2007). This suggests that not only is there variation between species, there may also be breed predispositions towards the presence of os cordis dextrum/sinistrum. More investigations into this possibility should be performed as this may correlate with other breed dispositions, especially cardiovascular disease.

### 2.18 Age and *Ossa cordis* correlations

*Ossa cordis* presence and size have been investigated using morphological and histological analysis, often comparing hearts of varying ages (Table 2). Cattle studies have shown that although the bone is present as a common feature, *Ossa cordis* size varied between two different age groups (<1 year and 1‐2 years); the os cordis dextrum length increased from 36.98 to 37.61 mm, respectively, and os cordis sinistrum lengths increased from 8.20 to 13.83 mm with age (Pour, 2004). In a one‐month‐old water buffalo calf, an os cordis was not observed; however, two *Ossa cordis* were present in all 14 individuals aged 3–5 years (Daghash & FarghaliI, 2017). Cardiac bone has been detected in adult sheep hearts but only cartilage was found in the heart of a four‐month‐old fetus (Frink & Merrick, 1974; Nabipour & Shahabodini, 2007). A distinct positive correlation was found between age and the length of *Ossa cordis* in deer, progressing from a mean length of 17.5 mm at 1.5 years old to 35.8 mm at 13.5 years old (Dupuy, 2011). In otters, evidence of bone and/or early ossification was observed in 12 hearts, including a juvenile and sub‐adult (Egerbacher et al., 2000). In 11 Doberman Pincher dog hearts, most presented with an os cordis, notably the 9‐week‐old puppies did not, whereas an 8.5‐week‐old individual did (James & Drake, 1968). In the adult population, one 3‐year‐old did not have an os cordis, but the remaining 7 dogs did. Cartilage was associated with 10 out of 11 cases, and a 3‐day‐old puppy did not have cartilage or bone present. In healthy horses’ cardiac cartilage does not typically develop until after 5 years of age, however, following cardiac arrhythmias, indicative of cardiac disease, a four‐year‐old horse presented with an os cordis (Matsuda et al., 2010). Generally, the presence and formation of *Ossa cordis* has been shown to be age‐dependent. In some species, *Ossa cordis* development appears to be a physiologically normal event and the bone develops over time. In contrast, cardiac disease could be a contributing factor regardless of age. Therefore, future investigations looking at hearts of varying ages, from more species is vital in understanding changes in the heart, due to both age and disease, which could be responsible for the formation and development of *Ossa cordis*.

### 2.19 *Ossa cordis* formation and development

It has been hypothesized that the *Ossa cordis* forms via endochondral ossification (EO), formation of bone tissue from cartilage, as opposed to intramembranous ossification, bone laid down directly onto mesenchyme (Egerbacher et al., 2000; Gopalakrishnan et al., 2007; Matsuda et al., 2010; Moittie et al., 2020). Currently, the evidence for EO formation is based mainly on the presence of cartilage associated with *Ossa cordis*. In buffaloes, the youngest heart investigated (30 days) had no os cordis but did have cartilage present, whilst older hearts (up to 5 years old) had *Ossa cordis* present in the same anatomical location as the cartilage observed in the calf, indicating that the bone may have developed from the cartilage (James & Drake, 1968). Similar findings were also indicated in deer where cartilage was present instead of bone in younger animals [<1.5 years; (Dupuy, 2011)]. Additionally, ossification of cartilage was seen histologically in eight of the 30 otter hearts investigated (Egerbacher et al., 2000). In the chimpanzee, three individuals with *Ossa cordis* had cartilage tissue attached to the bone, and in one other individual a C*artilago cordis* was observed in the same location as the *Ossa cordis* observed in the other specimens (Moittie et al., 2020). Additionally, well vascularized connective tissue surrounding the cartilage and cancellous (spongy) bone was observed in the camel (Balah et al., 2014) and the horse (Matsuda et al., 2010). In sheep, atrial bone formation was reported as being formed by EO (Gopalakrishnan et al., 2007), but an earlier study did not show any evidence of EO/cartilage (Frink & Merrick, 1974). The only species without direct evidence of EO are the cat, dog and cattle, however, investigations in dog and cattle hearts did find cartilage in close proximity to bone tissue, again indicating possible EO association (James, 1965; James & Drake, 1968; Liu et al., 1975). The theory of bone formation by EO is therefore supported by cartilage presence in many species with *Ossa cordis*. Naturally, evidence of cartilage in the same location as *Ossa cordis* (in say younger animals) or cartilage being associated with *Ossa cordis* is not direct evidence for EO. Indeed, many species contain a *Cartilago cordis* which do not appear to develop into an *Ossa cordis*. The lack of bone development in these species could be related to the size of the animal or heart shape. In otters, it was suggested that the reason for bone formation was related to unusually high mechanical forces on the heart for an animal of that size and its cone‐like heart shape (Egerbacher et al., 2000), indeed mechanical forces have been proven to affect the formation of cardiac tissues in general (Bishop & Lindahl, 1999). It is therefore possible that bone never develops in hamsters and other small species because they have a lower blood volume and therefore lower levels of forces within the heart required to pump a lower volume of blood, but it should be noted that smaller blood vessels might also increase resistance to flow. More investigations into *Ossa cordis* formation and cardiac forces are therefore required.

Interestingly, there is also very little knowledge pertaining to the initial formation of cartilage, which may eventually develop into *Ossa cordis*. In sheep atria, it has been suggested that during heart development, neural crest cells (NCCs) get lodged in the atrial wall and eventually differentiate into cartilage (Gopalakrishnan et al., 2007). This is supported by studies in the quail embryo where NCCs were observed differentiating into cartilage (Sumida, Akimoto, & Nakamura, 1989). It is possible cartilage in the heart forms from this, or other origins. One theory is that cardiac fibrocytes (involved in the formation of the cardiac skeleton, including cartilage) originate from epithelial to mesenchymal transformation (EMT). One study which investigated cardiac EMT stated that during embryonic formation every cell in the heart undergoes at least one EMT, so it is possible that EMT occurs in the formation of cardiac cartilage and subsequent bone (von Gise & Pu, 2012). Further research into the origins of cardiac cartilage, especially in relation to *Cartilago cordis* and *Ossa cordis*, with particular reference to NCCs and EMT, could elucidate the processes involved in *Ossa cordis* formation.

Interesting correlations could also be made between *Ossa cordis* and the equally elusive *Ossa genitalia* in mammals, as both are heterotopic bones, showing differing morphologies, in a limited number of species (Spani, Morigi, Bettuzzi, Scalici, & Carosi, 2020). The *Os penis* in rats and mice develops in two parts, the proximal and distal ends, using two different ossification processes. The distal part develops via membranous ossification, before fusing with the cartilaginous proximal part which later develops to bone by EO (Murakami & Mizuno, 1986). However, *Os penis* development is not consistent between species for example, whereas rodents use both membranous and endochondral ossification, the canine *Os penis* develops purely by EO (Nasoori, 2020). The differing *Os penis* developmental process shows us that despite evidence of EO in some specimens of *Ossa cordis*, this is no guarantee that EO is the only process involved in its development, and the process may vary between species.

### 2.20 *Ossa cordis* and cardiovascular disease correlations

A strong association was identified in chimpanzees between presence of *Ossa cordis* and idiopathic myocardial fibrosis (IMF). The study found that in the three hearts containing *Ossa cordis*, two had marked IMF (level 6) and one had moderate to severe IMF (level 5) (Moittie et al., 2020). The chimpanzee investigation hypothesized that tissue hypoxia may have prompted bone formation (Moittie et al., 2020). In chickens, cartilage formation was observed within fibrous tissue under low levels of oxygen delivery (ischemia), which in turn is often seen in cardiovascular disease (Lehoczky‐Mona & McCandless, 1964). *Ossa cordis* were found in canine diseased hearts, which presented with bundle of His degeneration caused by ischemia (James & Drake, 1968). It was proposed that the ischemia may have been worsened by the development of *Ossa cordis* gaining oxygen from a common arterial blood supply with the bundle. The common theme of ischemia throughout these publications may suggest that ischemia may cause *Ossa cordis* formation, and the cardiac condition worsens due to its presence. In 8.75% of people (*n* = 103 from 1177) the cardiac valves also contained metaplastic bone (Steiner, Kasparova, Kohout, & Dominik, 2007), this was hypothesized to be linked with diseases states and high mechanical forces.

Another theory is that bone forms in cardiovascular disease due to an abnormality in tissue repair (heterotrophic ossification), as indicated in the horse (Matsuda et al., 2010) although potential mechanisms were not provided. This theory is supported by incidental findings in rats (Aljinovic et al. 2016) focusing on left ventricular aneurysm repair. One of six rats had chondrocytes in the scar tissue which developed into bone via EO. However, the aneurysms were created by intentional ligation of a coronary artery and to our knowledge bone formation has not been observed in rats due to naturally occurring disease. It has been suggested that congenital cardiovascular disease may activate endothelial to mesenchymal transition (EndMT) causing endothelial cells to differentiate into bone or cartilage cells (von Gise & Pu, 2012). This work also showed that endothelial cell transition into fibroblasts and myofibroblasts resulted in cardiac fibrosis, similar to what has been seen in dogs with cardiac disease where *Ossa cordis* eventually developed (James & Drake, 1968). Therefore, *Ossa cordis* bone formation seen in cardiovascular disease‐affected animals, may form due to EndMT rather than directly due to ischemia or abnormal tissue repair.

One consequence of bone formation is that it may have detrimental effects on the hearts conduction system causing sudden death. In two children (aged 6 and 24 months) cartilaginous foci were found within the central fibrous body (Ferris & Aherne, 1971). The patients died suddenly, and it was suggested that cartilaginous foci may have caused dysfunction of the AVN, causing sudden death. In dogs with sudden, unexpected death, bone formation was present in eight of 11 cases and due to the proximity of os cordis to the bundle of His, it is possible that the bone had detrimental effects on the hearts conduction system (James & Drake, 1968). Further investigations into cats showed that os cordis or cartilaginous foci compressed the nodes of the heart causing lysis and granulation of nodular fibres (Liu et al., 1975). Sudden death in these cats might therefore be attributed to a lack of blood supply to the brain, caused by failing nodular activity within the heart.

In summary, cardiovascular disease seems to be associated with, or may even initiate, cardiac bone development, this may also be related to ischemia as a result of cardiovascular disease. Subsequently, once bone has formed, disease progression occurs either by causing more ischemia and/or by physical disruption of the conduction system of the heart. Whether disease progression results in sudden death, and the mechanisms employed, require further investigation, as do the initiation factors for bone formation via EO.

### 2.21 Proposed functions of *Ossa cordis*

Throughout the years, investigations into mammalian *Ossa cordis* have suggested two main theories in relation to the function of the cardiac bone(s). In cattle and camels, it has been proposed that the cardiac muscle anchors onto *Ossa cordis* improving contraction (James, 1965; Pour, 2004). In cattle, sheep and otters it has been suggested that *Ossa cordis* protect the heart from damage in areas of high mechanical stress during systole (Egerbacher et al., 2000; Frink & Merrick, 1974; James, 1965). Furthermore, in the chimpanzee, horse, cat and dog *Ossa cordis* presence was associated with cardiovascular disease and the overall function of *Ossa cordis* in these species was not specifically discussed (James & Drake, 1968; Liu et al., 1975; Matsuda et al., 2010; Moittie et al., 2020).

The anatomical position of *Ossa cordis* suggests they play mechanical roles in supporting the heart during contraction. In cattle, it has been noted that *Ossa cordis* act as fulcrums, similar to humans, in order to support the two atrioventricular valves during contraction (James, 1965; Pour, 2004). In humans, the fulcrum, a small tendinous structure within the heart, helps to anchor the myocardial band to aid contraction (Trainini et al., 2021). It is located in front of the aorta, just below the right trigone with the myocardial band originating from it and eventually, inserting back onto its point of origin. Histological analysis of human and camel hearts has also shown the presence of cardiac myocytes within the human fulcrum (Trainini et al., 2021) and within camel *Ossa cordis* and the surrounding cartilage (Hegazi, 1954). Moreover, it was indicated that the reason for this insertion into *Ossa cordis* could help stabilize hearts during contraction and relaxation. As cartilage and bone cells have been observed in the human fulcrum, this has drawn direct comparisons to *Ossa cordis* (Trainini et al., 2021). This contrasts to another investigation comparing bovine and human cardiac anatomy which argues that *Ossa cordis* may not be directly comparable to the human fulcrum, instead *Ossa cordis* may be more similar to the right trigone in humans (De Almeida et al., 2020). Despite these contrasting theories, it is possible that *Ossa cordis* have similar functions to the human fulcrum and may aid cardiac contraction.

A number of theories based on differing species have been suggested as to the roles and interactions between mechanical forces and *Ossa cordis* development. Formation of otter (or indeed other species) *Ossa cordis* from cartilage may have been triggered by mechanical stress (Egerbacher et al., 2000), similar to the generation of cardiac tissue via mechanical forces (Bishop & Lindahl, 1999). The anatomical location of *Ossa cordis* also supports this claim, as they frequently appear in the regions of the atrioventricular rings, between atrioventricular valves or even at the base of the aorta, areas of the heart which experiences the most mechanical pressure (Frink & Merrick, 1974; James, 1965). *Ossa cordis* do not usually form in utero, a time when mechanical forces are lower, it forms once the heart experiences more mechanical stress following birth. The sperm whale heart had no *Ossa cordis*, but they also generally lack of dense tissue in the heart (James et al., 1995), possibly as a result of the whale's buoyant habitat, thus resulting in significantly less mechanical stress. It is, therefore, reasonable to suggest that the entire pathway of *Ossa cordis* formation, which may include differentiation of NCCs and subsequent EO, could be triggered by mechanical stress, except of course in species lacking *Ossa cordis*. It is also worth stating that other than this one publication stating that *Ossa cordis* was not present in the whale, no other publication explicitly stated the absence of these bones in other species, and all papers stating their presence were discussed in this systematic review.

*Ossa cordis* may also play mechanical stress protective roles, preventing muscular damage in areas of high mechanical stress within the heart as part of its function. In cattle, the os cordis is found between the two atrioventricular valves (James, 1965). When both atrioventricular valves contract, they put a vast amount of force on the cardiac tissues between these valves therefore *Ossa cordis* may exist in order to prevent damage to these tissues (James, 1965). Research investigating camel hearts concurred with this theory, suggesting that the position of os cordis dextrum enables it to protect the heart during systole. The ovine os cordis sinistrum is also present in high‐stress areas between the aortic and the mitral valves, this is thought to help prevent shearing forces on sheep heart muscles (Frink & Merrick, 1974). However, the same study noted that the function of the os cordis dextrum was purely for additional support of the atrioventricular ring and not to protect muscular tissues.

The hypothesis that *Ossa cordis* play protective roles in the heart has also been used to help explain why *Ossa cordis* are present in some species and not others. For example, the peculiar shape of the otter heart causes more mechanical strain on heart tissues therefore cardiac bones may be present in the otter to protect the heart against these forces (Egerbacher et al., 2000). With age the heart is generally more prone to cardiovascular disease as the heart become less able to resist mechanical forces, this may explain why older animals have a higher prevalence of *Ossa cordis*.

*Ossa cordis* are well integrated into the conduction system, in close proximity to the atrioventricular node (AVN) and bundle of His (De Almeida et al., 2020; Frink & Merrick, 1974; James, 1965). In cattle, the AVN is only separated from the bone by a layer of adipose tissue (De Almeida et al., 2020; James, 1965). It is likely that the function of *Ossa cordis* is to aid in muscular contraction within the heart in addition to protecting vital components of the heart. By comparing *Ossa cordis* to an arguably better‐known structure, the human fulcrum, it is clear that the *Ossa cordis* likely play roles, at least in some species, in the contraction of the heart. In addition, the position and formation of *Ossa cordis* in relation to mechanical stresses indicates a role in the protection of vital cardiovascular structures. Alongside protecting against damage to muscular tissues and supporting areas of high mechanical stress, the close position of *Ossa cordis* to the conduction system of the heart suggests that the cardiac bone may play a vital role in protecting the AVN and bundle of His. Yet these theories do not adequately explain why some large, or indeed long living animals have *Ossa cordis*, whilst others do not.

## 3 CONCLUSIONS

*Ossa cordis* have a vast array of prevalence, numbers, morphologies and positions in differing individuals and between species. Variations may be due to differing bone development and formation, functions, stresses, pathologies and developmental stages of each animal. *Ossa cordis* may be required to aid in contraction or to protect vital components of the heart. The conditions surrounding *Ossa cordis* are those that it may be counteracting, for example high mechanical stress or cardiovascular disease, which in turn are the same conditions which may trigger bone formation in the first place. Therefore, the variation seen in *Ossa cordis* may be due to the variation in cardiac conditions which have changed between species during the course of evolution or indeed within an individual animal over its lifetime. The literature has suggested that *Ossa cordis* may also be detrimental to cardiac function. When presented together, it is difficult to assess whether cardiac disease or *Ossa cordis* independently compromise function, whether detrimental conditions caused by disease, for example ischemia, cause bone formation, and/or if pathological bone formation enhances disease progression.

In relation to *Ossa cordis* formation and development, evidence so far indicates it may be initiated via EO originating from cardiac cartilage. If this is the case, why do most species with *Cartilago cordis* not then develop *Ossa cordis*? Are pathways and mechanisms activated or prevented in these species to prevent bone formation or vice versa in species which do develop *Ossa cordis*? Certain risk factors such as age or cardiac conditions may trigger EO as these conditions develop denser tissue in the heart, yet in some species *Ossa cordis* are present in healthy, young hearts. It is also very likely that genetic lines/breeding and evolutionary pathways play roles in *Ossa cordis* formation. Further research is needed to further understand the anatomy, histology, functions, risk factors, pathologies and mechanisms related to *Ossa cordis*.

## CONFLICT OF INTEREST

The authors declare no competing interest.

## Supporting information


**FIGURE S1** PRISMA chart showing references included within the systematic review. Used the method detailed in the literature (Page et al., 2021).

**Video I** Chimpanzee *Ossa cordis*. A 3D model of the specimen rendered from high‐resolution X‐ray computed microtomography data using VGStudioMAX v2.2 software (https://www.volumegraphics.com). Samples and methods used were as described previously (Keane, Paul, Sturrock, Rauch, & Rutland, 2017; Moittie et al., 2020).

## Data Availability

The data that support the findings of this study are available from the corresponding author upon reasonable request.
