# Supplementary figures and images for: Anatomy, histology, development and functions of Ossa cordis: A review
Source: Anat Histol Embryol. 2022 Sep 8;51(6):683–95. doi: 10.1111/ahe.12861 (PMC9826330; doi:10.1111/ahe.12861)

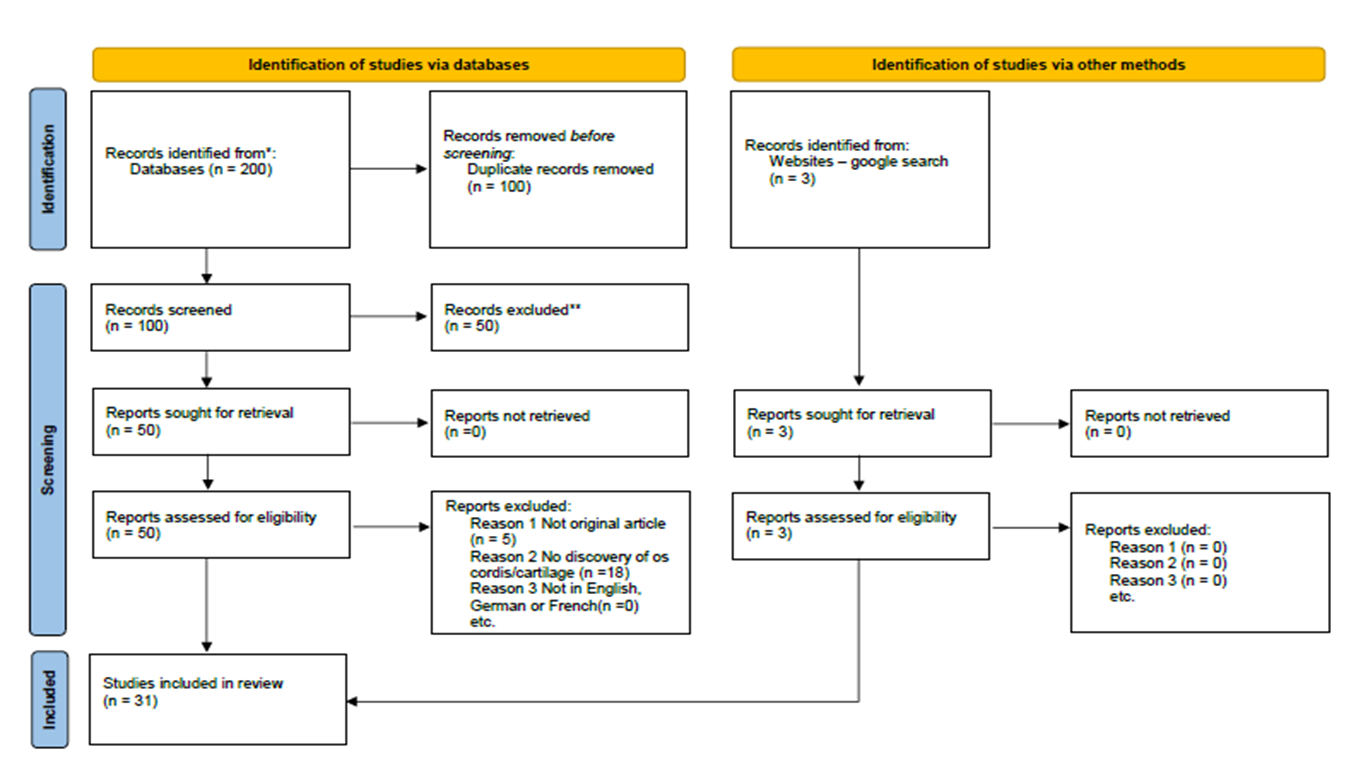

Supplement: Supplementary file 2 — Video I Chimpanzee Ossa cordis. A 3D model of the specimen rendered from high‐resolution X‐ray computed microtomography data using VGStudioMAX v2.2 software (https://www.volumegraphics.com). Samples and methods used were as described previously (Keane, Paul, Sturrock, Rauch, & Rutland, 2017; Moittie et al., 2020). [file AHE-51-683-s001.jpg]
